# The Evolutionary Ecology of Biotic Association in a Megadiverse Bivalve Superfamily: Sponsorship Required for Permanent Residency in Sediment

**DOI:** 10.1371/journal.pone.0042121

**Published:** 2012-08-08

**Authors:** Jingchun Li, Diarmaid Ó Foighil, Peter Middelfart

**Affiliations:** 1 Museum of Zoology, Department of Ecology and Evolutionary Biology, The University of Michigan, Ann Arbor, Michigan, United States of America; 2 Australian Museum, Sydney, New South Wales, Australia; Brigham Young University, United States of America

## Abstract

**Background:**

Marine lineage diversification is shaped by the interaction of biotic and abiotic factors but our understanding of their relative roles is underdeveloped. The megadiverse bivalve superfamily Galeommatoidea represents a promising study system to address this issue. It is composed of small-bodied clams that are either free-living or have commensal associations with invertebrate hosts. To test if the evolution of this lifestyle dichotomy is correlated with specific ecologies, we have performed a statistical analysis on the lifestyle and habitat preference of 121 species based on 90 source documents.

**Methodology/Principal Findings:**

Galeommatoidea has significant diversity in the two primary benthic habitats: hard- and soft-bottoms. Hard-bottom dwellers are overwhelmingly free-living, typically hidden within crevices of rocks/coral heads/encrusting epifauna. In contrast, species in soft-bottom habitats are almost exclusively infaunal commensals. These infaunal biotic associations may involve direct attachment to a host, or clustering around its tube/burrow, but all commensals locate within the oxygenated sediment envelope produced by the host’s bioturbation.

**Conclusions/Significance:**

The formation of commensal associations by galeommatoidean clams is robustly correlated with an abiotic environmental setting: living in sediments (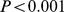). Sediment-dwelling bivalves are exposed to intense predation pressure that drops markedly with depth of burial. Commensal galeommatoideans routinely attain depth refuges many times their body lengths, independent of siphonal investment, by virtue of their host’s burrowing and bioturbation. In effect, they use their much larger hosts as giant auto-irrigating siphon substitutes. The evolution of biotic associations with infaunal bioturbating hosts may have been a prerequisite for the diversification of Galeommatoidea in sediments and has likely been a key factor in the success of this exceptionally diverse bivalve superfamily.

## Introduction

One of the classic questions in biology concerns the mechanisms that control the generation and maintenance of planetary biodiversity [1]. Two broad classes of macroevolutionary drivers are generally recognized. The Red Queen model [2], [3] states that biotic factors play major roles in shaping lineage diversification, while the Court Jester model [4] places more emphasis on abiotic factors. Although both sets of drivers operate on different spatial and temporal scales [4], they clearly play off each other [5] and their relative importance remains an active area of contention in fundamental biodiversity research [6]–[8].

The importance of biotic drivers is most evident in terrestrial ecosystems whose dominance by insects and angiosperms is attributed substantially to coevolutionary dynamics [9]. Much of the evidence for biotic drivers of marine diversification is paleontological [6], [10]–[12] and, with some notable exceptions (*e.g.*, [13], [14]), neontological marine evolutionary studies typically focus on abiotic drivers [15]–[18]. This is primarily because the scope of ecological interactions remains poorly characterized for most marine clades, especially regarding subtle effects such as facilitation (presence of one species enhances survival of another) that may be very important in nature [5], [9]. Our ignorance concerning the role of biotic interactions in macroevolutionary processes is being increasingly recognized as a serious deficiency that may underlay the frequent mismatch between empirical data and theoretical models [5]–[7], [20]. Given this, how might one test the relative importance of marine biotic and abiotic diversification drivers in an extant marine clade?

Our approach is comparative and involves targeting an exemplar marine taxon, the marine bivalve superfamily Galeommatoidea. This clade is suitable for addressing our question for two reasons. Firstly, Galeommatoidea is recognized as a “megadiverse” group [21]. Those small-bodied (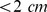) bivalves comprise an estimated 500 described species [22], although this is a serious underestimate: a large fraction remains undescribed [21], [23]. Recent quantitative biodiversity surveys of Western Pacific coral reefs have found that Galeommatoidea had the highest species diversity among Bivalvia, despite their relatively low abundance [21], [24]. Secondly, Galeommatoidea embodies a clear ecological dichotomy in that some members are free-living while others have obligate biotic associations (mostly commensals) with invertebrate hosts [25], [26]. The commensals exhibit specific host-taxes [27]–[30], although in some cases commensals may associate with multiple hosts [29], [31], [32] and single host species may be colonized by multiple commensals [33], [34].

Our strategic goals are to test the relative importance of free-living and commensal life styles in driving galeommatoidean diversification and to establish the ecological context for evolutionary transitions among the two life styles. The former goal involves constructing comprehensive phylogenetic trees that will allow us to detect the effect of the traits of interest (presence/absence of biotic association) on diversification rates. In this present study, our focus is on the latter goal. If the lifestyle dichotomy is correlated with discrete ecologies, specific hypotheses regarding the role of facilitative biotic associations can be proposed and tested.

Galeommatoidea has significant diversity in the two primary benthic habitats: soft- and hard-bottoms. The two types of habitats differ greatly in terms of physical properties as well as in faunal composition and community structure [35]–[37]. Adaptation to either habitat requires a certain degree of morphological and behavioral specialization [35]. Previous workers have hypothesized that commensalism in Galeommatoidea is an adaptation to soft-bottom infaunal habitats [25], [38], but this hypothesis has not been formally tested at the superfamily level. We do so here by performing a literature based statistical analysis to test if the evolution of this pronounced lifestyle dichotomy is correlated with the acquisition of discrete benthic ecologies.

## Results and Discussion

**Figure 1:**
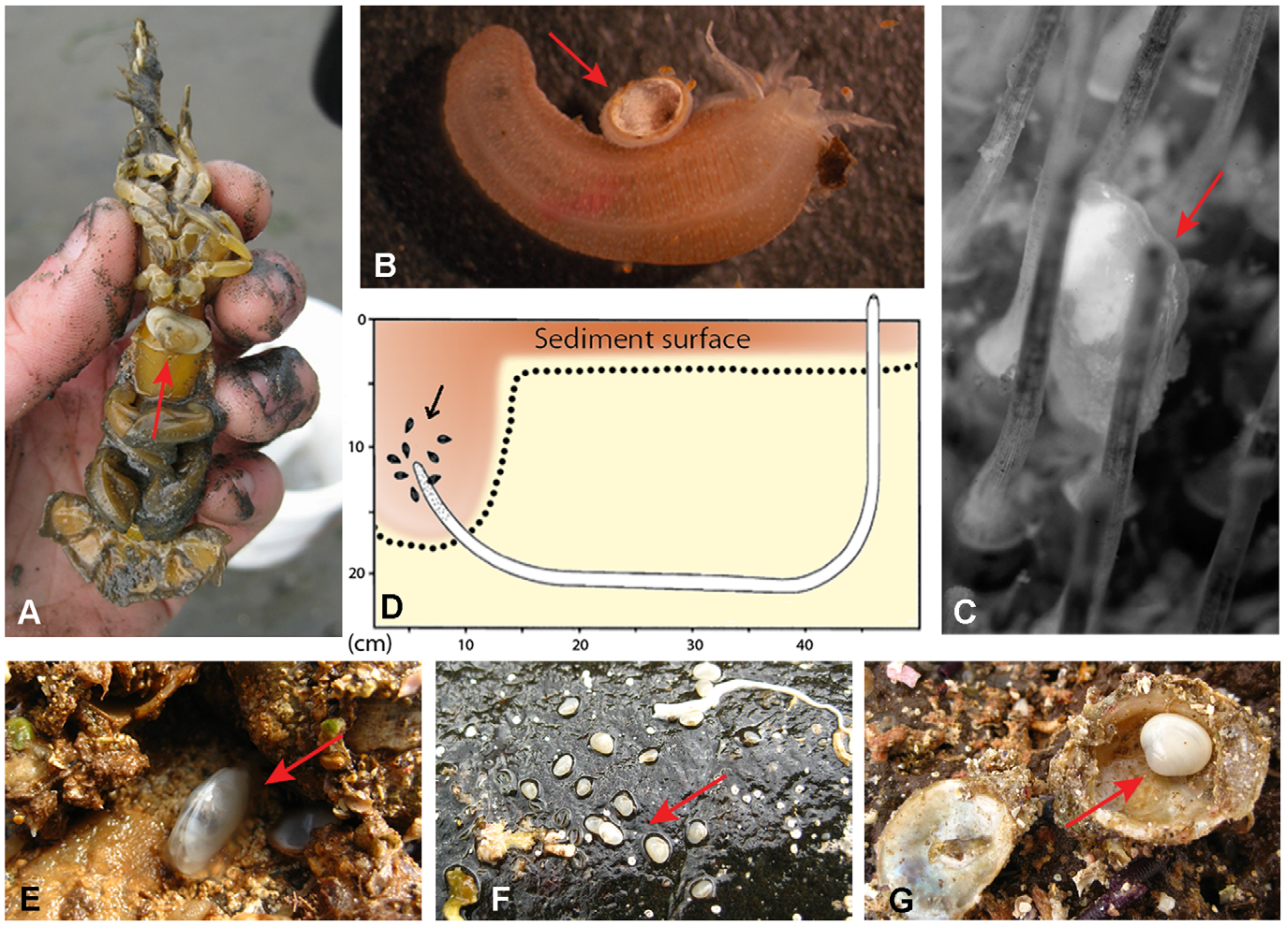
Exemplar commensal and free-living galeommatoideans. A. The commensal clam *Neaeromya rugifera* attached to the ventral side of a mud shrimp *Upogebia pugettensis*. B. The commensal clam *Scintillona bellerophon* attached to its holothuroid host *Leptosynapta clarki*. C. The commensal clam *Waldo sp.* attaching to the surface of its benthic irregular sea urchin host *Brisaster latifrons*. D. Clustering of commensal *Rochfortia* (*Mysella*) *tumida* (arrow), within the exhalent oxic halo of *Mesochaetopterus taylori*. Dotted line separates oxygenated (red) and anoxic (yellow) sediment zones (After [62]). E. The free-living *Scintilla* (*Lactemiles*) *strangei* in its rock crevice. F. Underside of a rock showing several free-living *Borniola lepida* individuals attached by byssal threads. G. A free-living *Kellia sp.* nestled within an empty bivalve shell. (Photo credit: A, E–G: J. Li; B: L. Kirkendale; C: D. Ó Foighil).

Habitat and life-style information for 121 galeommatoidean species was extracted from the literature (see Table S1 for details) and the Materials and Methods section summarizes how case studies were classified as being free-living, commensal or (facultatively) both. Our dataset encompassed representatives from all major ocean basins and from a wide variety of benthic habitats. It contained a total of 57 free-living taxa, *i.e.*, occupying abiotic microhabitats (Fig. 1E, F, G ) and 60 commensal species. Many of the commensals directly attached to their invertebrate hosts (Fig. 1A, B, C), the remainder locating around host tubes/burrows (Fig. 1D). We also obtained data on 4 species with facultative lifestyles that were reliably recorded from abiotic as well as biotic microhabitats.

Our main result is presented in Table 1: commensal and free-living galeommatoidean taxa exhibited a striking ecological disjunction in benthic habitat type. All but 2 of 57 free-living species were restricted to hard-bottom habitats, typically hidden in rock/coral crevices. In contrast, 56 out of 60 commensal species were infaunal sediment dwellers. Our result establishes that formation of commensal associations by galeommatoidean clams is robustly correlated with living in sediments (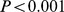). This clear-cut finding is consistent with the hypothesis that biotic association is primarily an adaptation to living in soft-bottom infaunal habitats [25], [38], but does not, in itself, explain the putative adaptive nature of such associations.

**Table 1:** Numbers of species that belong to each habitat-lifestyle combination.

	Free-living	Commensal	Both	Total
**Hard-bottom**	55	4	2	61
**Soft-bottom**	2	56	2	60
**Total**	57	60	4	121

### Soft-Bottom Taxa

How might we test the adaptive significance of biotic association in sediment-dwelling Galeommatoidea? One approach would be to perform detailed comparative ecological studies of fitness in species that have facultative life styles and contain significant numbers of free-living and commensal individuals. Two of the four facultative life style taxa in our survey occur in sediments: *Kurtiella bidentata* (Montagu, 1803) and *Mysella vitrea* (Laseron, 1956) [29], [39], [40], and the ecology of the former has been studied in considerable detail. *K. bidentata* is associated with an unusually wide variety of bioturbating invertebrate hosts, most notably with the burrowing ophiuroid *Amphiura filiformis* [29]. Across its range, commensal individuals of *K. bidentata* attain much greater population densities [29], [39] and locate deeper in the sediment [29], [39], [41] (Table 2) than do free-living conspecifics. These distinctions have been attributed to two very different processes. One hypothesis states that positioning of commensals within the hosts’s oxygenated burrow provides a depth refuge from predation and that the increased commensal population density stems from lower mortality rates [29]. A competing hypothesis views *K. bidentata*’s commensal associations as byproducts of density-dependent competition: high population densities driving individuals deeper into the sediment to form commensal associations [39]. Available evidence strongly favors the predation depth refuge hypothesis: *K. bidentata* exhibits positive host chemotaxis irrespective of clam density and free-living populations do experience much higher mortality rates (and lower fitness) than commensals [29].

**Table 2:** Habitat depth of selected soft-bottom galeommatoideans (free-living examplers are indicated).

Species	Habitat depth	Max. shell length	References
*Mysella charcoti* (free)	Top few millimeters	3.0 mm	[54]
*Mysella narchii* (free)	Top few millimeters	3.1 mm	[88]
*Kurtiella bidentata* (host absent)	0–5 cm	3.5 mm	[29], [39]
*Kurtiella bidentata* (host present)	5–50 cm	3.5 mm	[29], [39]
*Mysella vitrea* (host present)	15–95 cm	5 mm	[40]
*Arthritica bifurca*	about 6 cm	4.1 mm	[30], [86]
*Brachiomya stigmatica*	10–15 cm	3.0 mm	[33]
*Divariscintilla maoria*	over 15 cm	6.0 mm	[89]
*Halcampicola tenacis*	15–30 cm	5.0 mm	[90]
*Montacuta elevata*	up to 17 cm	6.0 mm	[53]
*Montacutella echinophila*	10–15 cm	7.9 mm	[33]
*Nipponomysella subtruncata*	5–15 cm	6.8 mm	[91]
*Rochfortia* (*Mysella*) *tumida*	12–15 cm	3.5 mm	[62]

Predation is a key factor that affects species survival and community structure in benthic environments [42]–[44] and bivalves have evolved two general anti-predator strategies: increasing handling time (via armor) or reducing the encounter rate (via avoidance) [45]. Galeommatoideans are small-bodied clams that typically specialize in avoidance rather than armor; indeed many species (in both hard- and soft-bottom substrates) have undergone significant shell reduction and/or internalization [23], [25], [46]. In hard bottom substrates, crevices provide preexisting spatial refuges. Crevices are not available in soft-bottom substrates and the most common avoidance adaptation is to become infaunal [45]. The depth refuge hypothesis for *Kurtiella bidentata* [29] is consistent with extensive experimental evidence that predation pressure on infaunal bivalves drops markedly with depth of burial [45], [47]–[52].

What about the rest of the soft-bottom Galeommatoidea? Although the data are limited, commensalism is typically associated with deeper burial. For instance, the other facultative species, *Mysella vitrea*, positions significantly deeper in sediments in the presence of its host [40] and recorded depths for most commensals are much deeper than the two known free-living sediment dwellers, the Antarctic species *M. charcoti* and *M. narchii*, which are restricted to the top few millimeters of sediment (Table 2). The few data on predation rates includes reports of greatly reduced predation on the deeply buried commensal *Aligena elevata* [53] but heavy predation on the shallowly buried non-commensal *M. charcoti* [54]. *M. charcoti* survives passage through the alimentary tracts of some predatory fishes, and may indeed be dispersed primarily through this process [55], indicating that in this non-commensal species armor rather than avoidance may be the primary anti-predation strategy. Why this strategy is not more widely adopted by non-Antarctic galeommatoideans is not clear, but may be related to a greater spectrum of shell-crushing/boring/disarticulating predators operating on temperate and tropical sediment-dwellers.

Predator avoidance through deeper burial is not cost-free because the infauna requires contact with the sediment-water interface for basic physiological functions including respiration, and in many cases also feeding, reproduction and defecation [56]. Most infaunal bivalve species engage in a trade-off between access to the interface and lethal predator avoidance by investing in extendable siphons that allow individuals to directly contact the water column while their main body mass remains deeply buried. Burial depth is therefore a function of siphon length and biomass, but the clams are still exposed to sub-lethal predation on exposed siphon tips [48], [57]–[59]. In contrast, most galeommatoidean bivalves have modest siphons or even lack them completely [25], [26], yet commensal species routinely attain sediment depth refuges many times their body lengths (Table 2).

Within-sediment galeommatoidean hosts are bioturbators that construct irrigated tubes/burrows. Bioirrigation and bioturbation processes facilitate nutrient intake from the water column and oxygen penetration into deeper sediment [60], [61]. By locating within the host’s oxygenated sediment envelope [29], [38], [62], commensal galeommatoideans in effect use their much larger hosts as giant auto-irrigating siphon substitutes. This enables commensals to decouple burial depth from body size and solve the surface access/predator avoidance trade-off while remaining small-bodied; other benefits such as filter-feeding from respiration or feeding currents of the hosts could also accrue. The scope of depth refuges obtained by commensal galeommatoideans is set by host borrowing parameters and spans that of free-living infaunal bivalves. For instance, the world’s largest burrowing clam, recently renamed *Panopea generosa* [63], attains a depth refuge of up to 1 meter below the sediment/water column interface thanks to its enormous siphons [64]. Remarkably, this maximum burial depth is matched by the tiny (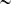
5 mm in body length) facultative commensal *Mysella vitrea* in sediments bioirrigated by its host, the ghost shrimp *Trypaea australiensis* [40].

### Hard-Bottom Taxa

The vast majority of hard-bottom species are free-living (Table 1). They nestle in crevices within or underneath rocks, coral heads or encrusting epifauna that are passively ventilated by ambient water flow [46] and they may show a simple hierarchy of geo-, photo- and thigmotaxes to remain within these microhabitats [65]. Unlike sediments, crevices are common in hard-bottom benthos and afford these minute clams effective abiotic refuges from predators in addition to contact with the water column [46], [66]. With the possible exception of *Pristes oblongus*, a poorly studied species reported to attach to chitons [67], the relatively small number of hard-bottom commensals all associate with infaunal hosts that can form burrows in hard substrates. They include *Arthritica crassiformis* associated with the boring bivalve *Anchomasa similis* [68]; *Ephippodonta lunata* and *Ephippodontana macdougalli* in the burrow of slow shrimp *Strahlaxius plectorhynchus* [69], and the genus *Jousseaumia* associated with sipunculans within corals [70]. Note that *Ephippodonta lunata* and *Ephippodontana macdougalli* are facultative commensals that are also found in rock crevices [69], but we have no data on comparative survival rates of free-living and commensal individuals. It is likely that abiotic crevices in most hard-bottom benthic environments greatly exceed, in number and in spatial heterogeneity, those produced by any actual or potential host species. The overwhelming predominance of free-living galeommatoidean lifestyles in these communities (Table 1) suggests that for this bivalve superfamily, the number of available crevices is more important than crevice spatial uniformity, or biotic association, in promoting lineage diversification in hard-bottom benthic environments.

### Biotic Association and Diversification

Infaunal sediment bioturbators have long been recognized as key ecosystem engineers that alter the physical and chemical properties of the substrate and impact nutrient cycles [71]–[73]. Their biotic impact on benthic communities is also an active topic area in both paleontological macroevolutionary [5], [73], [74] and neontological microevolutionary [61], [75] studies. It is typically negative for co-occurring taxa that require stable sediments, but positive, over both ecological and evolutionary timescales, for commensal species [5], [61]. This latter effect is robustly evident for galeommatoideans and our data strongly support the hypothesis that formation of commensal relationships with burrowing macroinvertebrates has been a key adaptation in their success in sediments [25], [38]. This is significant because most of the global marine benthos is soft bottom [76], [77] and relatively few bivalve lineages (*e.g.*, Mytilidae [78], Pectinidae [79] and Arcoidea [80]) have achieved significant diversity in both hard-bottom and soft-bottom habitats, presumably due to the distinctive functional/morphological constraints imposed by adapting to either habitat [81]. Sediment-dwelling Galeommatoidea have superseded these functional/morphological constraints via behavioral innovation; acquiring many of the necessary functions, including deep burrow construction and irrigation, indirectly through biotic association with larger invertebrate infauna.

Our literature survey returned an approximately equal number of soft- and hard-bottom galeommatoidean species (Table 1), although the true ratio is unknown due to the very significant number of undescribed species in both habitats [21], [23], [46]. Nevertheless, it is clear that commensalism underlies the evolutionary genesis of a major fraction of galeommatoidean diversity and has likely been instrumental in attaining their “megadiverse” status among marine bivalves [21]. Unlike most bivalve lineages, Galeommatoidea does not have a comprehensive fossil record for effectively inferring its long-term diversity dynamics. In fact, less than half of the living genera are known from the fossil record [82]. Therefore, an in-depth understanding of the role that biotic association has played in galeommatoidean diversification requires a detailed molecular phylogenetic framework for the group. This is currently unavailable, but is badly needed as there is very little consensus regarding supra-specific taxonomic relationships in this superfamily [21], [23], [26], [83], [84]. The Red Queen and Court Jester models provide a simple theoretical framework: do commensal galeommatoideans represent discrete adaptive radiations where speciation is driven by host-shifts (Red Queen) or a polyphyletic melange of evolutionary dead-ends (Court Jester)? We are presently constructing molecular phylogenies to address these questions.

### Conclusions

Evolutionary studies of contemporary marine biotas are typically framed within abiotic hypothesis-testing contexts and have collectively lagged behind terrestrial studies in developing an integrated framework that includes a meaningful biotic/ecological perspective. The strong correlation between lifestyle and habitat preference in Galeommatoidea suggests that the relative importance of the Red Queen model can be greatly influenced by abiotic ecological factors such as benthic substrate type: maximal in soft-bottom and minimal in hard-bottom. Facilitative biotic associations such as commensalism are not rare in marine environments [85], and it is likely that the evolution of many other commensal-rich marine benthic lineages have also been tailored by ambient abiotic factors.

## Materials and Methods

To investigate whether commensal life styles in galeommatoidean clams are correlated with specific benthic habitat types, we extracted habitat and lifestyle information for a total of 121 species from 90 source documents, including peer-reviewed journals, book chapters, museum report and personal observations (see all references in Table S1). Our data set contains a number of likely sampling biases. Due to limitations in marine sampling methodologies, our species pool is weighted toward taxa from intertidal and shallow subtidal habitats and there is a relatively low representation of deep-sea taxa. However this is unlikely to affect our results because the sampling bias applies to both hard-bottom and soft-bottom deep-sea species. A potentially more serious bias could involve significant differences in sampling free-living versus commensal sediment dwellers. If the former were relatively intractable, it would bias our results in favor of the hypothesis. We consider this unlikely, however, because free-living taxa are easier to sample given their primary location in the shallow surface layers of sediment, rather than in the deep burrows of their commensal hosts.

### Searching

The initial literature search was conducted through the ISI Web of Knowledge database using “Galeommatoidea” as a topic keyword. This search resulted in 57 English publications between the years of 1899 and 2011. Because much of the relevant literature on this superfamily is not archived in the ISI web of Knowledge database, we investigated the older literature cited by these 57 publications and elicited additional sources from The Australian Museum Research Library and The University of Michigan Museums Library. These activities yielded an additional 69 publications to give a total of 126.

### Selection

Our classification criteria for habitat and lifestyle data were as follows. Benthic habitat was divided into two major categories: soft-bottom and hard-bottom. Soft-bottom includes all benthic substrates composed of unconsolidated sediment, whereas hard-bottom includes all rocky or consolidated substrates, including coral galleries. Lifestyle was classified as either commensal, free-living or (facultatively) both. To obtain a “commensal” designation, taxa had to have identified hosts; a generic assumption of a commensal lifestyle by the reporting authors was insufficient. Host identification can be relatively straightforward in cases where the commensal galeommatoidean attaches directly to its host (Fig. 1A, B, C) and is not dislocated during sampling. In contrast, it can be quite difficult when the commensal remains unattached and locates in the oxygenated envelope surrounding its host’s temporary burrow (Fig. 1D). In the latter cases, it may require very careful benthic sampling, and/or laboratory behavioral experiments, to identify specific host taxa [29], [38]. We encountered a few cases of galeommatoidean taxa that were initially listed as free-living, prior to subsequent host identification, *e. g. Arthritica bifurca* [30], [86]. In addition, a small number of species were reliably recorded as being both commensal and free-living. These were classified as facultative commensals.

### Validity Assessment

Critical analysis of these 126 publications found 36 to be deficient in that they contained insufficient information to unambiguously determine habitat (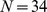) or lifestyle (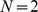) of the species of interest. All 36 were removed from the analysis, resulting in a final working list of 90 publications. Excluding 2 putatively commensal galeommatoidean species with unidentified hosts may have resulted in an underestimation of the relative number of commensal taxa. However, all of these excluded putative commensal occurred in soft-bottom benthic habitats and their exclusion has therefore not contributed to the pronounced correlation of commensalism and sediment-dwelling observed in the 60 commensal taxa analyzed.

### Data Abstraction

Galeomatoidean habitat type and life style information was extracted, identified and classified manually for a total of 121 species from our final list of 90 publications (see Table S1 for detailed habitat and lifestyle information for all species included). The numbers of species that belonged to each habitat-lifestyle combination were summarized in a contingency table (Table 1).

### Quantitative Data Synthesis

In order to detect possible correlations between habitat preference and lifestyle, Fisher’s exact test was performed using R 2.13.1 [87]. Note that a small number of facultative (*i.e.*, both commensal and free-living) species are present in the table, but these were not included in the test because it is inappropriate to classify them discretely as either commensal or free-living.

## Supporting Information

Table S1 Available galeommatoidean habitat, lifestyle and (for commensal species) host information, including references.
(PDF)
