# Supplementary material for: The Evolutionary Ecology of Biotic Association in a Megadiverse Bivalve Superfamily: Sponsorship Required for Permanent Residency in Sediment
Source: PLoS One. 2012 Aug 8;7(8):e42121. doi: 10.1371/journal.pone.0042121 (PMC3414514; doi:10.1371/journal.pone.0042121)
Supplement: Table S1 — Available galeommatoidean habitat, lifestyle and (for commensal species) host information, including references. (PDF) [file pone.0042121.s001.pdf]

Soft-bottom	S	Commensal	C
Hard-bottom	H	Free-living	F

Available galeommatoidean habitat, lifestyle and (for commensal species) host information, including references
Species names are arranged in alphabetical order

Species			Hosts	Habitat Details	References
Anisodevonia ohshimai	S	C	*Patinapta ooplax*	Attached to host	Kato, 1998; Kawahara, 1942
Arthritica bifurca	S	C	*Pectinaria australis*	Attached to host tubes	Chanley and Chanley, 1980<br>Wear, 1966
Arthritica crassiformis	H	C	*Barnea similis*	Attached to host (rock-boring pholadid)	Chanley and Chanley, 1980; Morton, 1973; Ponder, 1965
Arthritica japonica	S	C	*Xenophthalmus pinnotheroides*	Attached to host	Lützen *et al.*, 2003
Austrodevonia sharnae	S	C	*Taeonigyrus australianus*	Attached to host	Middelfart and Craig, 2004
Barrimysia siphonosomae	S	C	*Siphonosoma cumanense*	In host burrows	Jespersen *et al.*, 2002; Morton and Scott, 1989
Borniola lepida	H	F		Under rocks	Personal observation, Li, 2011
Brachiomya stigmatica	S	C	*Brissus latecarinatus*	Attached to host	Jespersen *et al.*, 2004; Yamamoto and Habe, 1974
Chlamydoconcha orcutti	H	F		Under large stones	Morton, 1981
Curvemysella paula	S	C	*Spiropagurus spiriger; Diogenes edwaedsii*	Within host-occupied shells	Goto *et al.*, 2007
Devonia perrieri	S	C	*Leptosynapta inhaerens*	Attached to host	Clench and Aguayo, 1931
Divariscintilla cordiformis	S	C	*Lysiosquilla scabricauda*	In host burrows	Mikkelsen and Bieler, 1992
Divariscintilla luteocrinita	S	C	*Lysiosquilla scabricauda*	In host burrows	Mikkelsen and Bieler, 1992
Divariscintilla maoria	S	C	*Lysiosquilla spinosa*	In host burrows	Judd, 1971
Divariscintilla octotentaculata	S	C	*Lysiosquilla scabricauda*	In host burrows	Mikkelsen and Bieler, 1992
Divariscintilla troglodytes	S	C	*Lysiosquilla scabricauda*	In host burrows	Mikkelsen and Bieler, 1989
Divariscintilla yoyo	S	C	*Lysiosquilla scabricauda*	In host burrows	Mikkelsen and Bieler, 1989
Duoconclavis piscator	H	F		Under stones	Middelfart, 2005
Entovalva amboinensis	S	C	*Patinapta laevis*	Within host esophagus	Bristow *et al.*, 2010; Spärck, 1931
Entovalva lessonothuriae	S	C	*Holothuria (Lessonothuria) pardalis*	Within host esophagus	Kato, 1998
Entovalva major	S	C	*Holothuria curiosa*	Attached to host	Lützen *et al.*, 2005
Entovalva mirabilis	S	C	*Patinapta crosslandi*	Within host esophagus	Bristow *et al.*, 2010; Voeltzkow, 1890
Entovalva nhatrangensis	S	C	*Holothuria leucospilota; Holothuria spinifera*	Within host esophagus	Bristow *et al.*, 2010
Entovalva semperi	S	C	*Protankyra bidentata*	Attached to host	Morton and Scott, 1989, Ohshima, 1930
Ephippodonta gigas	H	F		Under boulders	Kubo, 1996; Lützen and Nielsen, 2005
Ephippodonta gregaria	H	F		In crevices	Gofas, 1991; Middelfart, 2005
Ephippodonta lunata	H	C	*Strahlaxius plectorhynchus*	In host burrows	Cotton 1938; Middelfart, 2005
Ephippodonta lunata	H	F		In rock crevices	Cotton 1938; Middelfart, 2005
Ephippodontina murakamii	H	F		Attached to deep sea coral	Arakawa, 1960; Middelfart, 2005
Ephippodontina oedipus	H	F		Within coral galleries	Middelfart, 2005
Ephippodontoana macdougalli	H	C	*Strahlaxius plectorhynchus*	In host burrows	Cotton 1938; Middelfart, 2005
Ephippodontoana macdougalli	H	F		In rock crevices	Cotton 1938; Middelfart, 2005
Ephippodontomorpha hirsutus	S	C	*Lysiosquillina maculata or L. tredecimentata*	In host burrows	Middelfart, 2005
Epilepton clarkiae	S	C	*Various sipunculans*	Attached to host	Jespersen *et al.*, 2007
Fronsella ohshimai	S	C	*Sipunculus nudus*	Attached to host	Manning and Morton, 1987
Galeomma ambigua	H	F		Under shale blocks	Lützen and Nielsen, 2005
Galeomma coalita	H	F		Under stones	Gofas, 1991
Galeomma layardi	H	F		Under rocks	Lützen and Nielsen, 2005
Galeomma obockensis	H	F		Under coral blocks	Lützen and Nielsen, 2005
Galeomma phuketi	H	F		Under shale blocks	Lützen and Nielsen, 2005
Galeomma sagenata	H	F		In coral rubble	Oliver and Holmes, 2004
Galeomma takii	H	F		Under rocks	Morton, 1973b
Galeomma turtoni	H	F		In large crevices	Gofass, 1991
Halcampicola tenacis	S	C	*Halcampoides sp.*	Attached to host	Oliver, 1993
Jousseaumlella heterocyathi	H	C	*Aspidosiphon sp.*	Attached to host within coral crevices	Bourne, 1906
Jousseaumlella heteropsammiae	H	C	*Aspidosiphon sp.*	Attached to host within coral crevices	Bourne, 1906
Kellia jacksoniana	H	F		In crevices, within mussel beds	Laseron, 1956
Kellia laperousii	H	F		In crevices, inside empty shells	Keep and Hannibal, 1911

Kellia porculus	H	F		Within coral galleries	Morton and Scott, 1989
Kellia suborbicularis	H	F		In crevices, inside empty shells	Jespersen and Lützen, 2007; Lebour, 2006
Koreamya arcuata	S	C	*Lingula anatina*	Attached to host	Lützen *et al.*, 2009; Sato *et al.*, 2011
Kurtiella bidentata	S	C	*Amphiura filiformis; Maxmuelleria lankesteri; Nephtys incisa*	In host burrows	Jespersen and Lützen, 2001; Nickell *et al.*, 1994; Ockelmann and Muus, 1978; Prevedelli *et al.* 2001
Kurtiella bidentata	S	F		Upper sediment layer	Prevedelli *et al.* 2001
Kurtiella pellucida	H	F		Within bioclastic gravels	Gofas and Salas, 2008
Kurtiella triangularis	H	F		In rock and algal turf crevices	Gofas and Salas, 2008
Lasaea adansoni	H	F		In rock crevices	Altnöder and Haszprunar, 2008; Crisp and Standen, 1988
Lasaea australis	H	F		In rock and encrusting epifaunal crevices	Ó Foighil & Thiriot-Quievreux, 1999
Lasaea colmani	H	F		In rock and encrusting epifaunal crevices	Ó Foighil & Thiriot-Quievreux, 1999
Lasaea maoria	H	F		In damp crevices and beneath stones	Ponder, 1971b
Lasaea undulata	H	F		In rock and encrusting epifaunal crevices	Iwasaki, 1996
Lepton squamosum	S	C	*Upogebia deltaura; Upogebia stellata*	In host burrows	Kallonas *et al.*, 1999; Norman *et al.*, 1891
Litigiella glabra	S	C	*Sipunculus nudus*	Attached to host	Kallonas *et al.*, 1999; Lamy, 1908
Marikellia solida	H	F		Crevices, within mussel beds	Laseron, 1956
Melliteryx acupuncta	H	F		Under rocks	Personal observation
Montacuta percompressa	S	C	*Leptosynapta tenuis*	Attached to host	Chanley and Chanley, 1970; Fox *et al.*, 2007
Montacuta phascolionis	S	C	*Phascolion strombi*	Within host-occupied shells	Jespersen and Lützen, 2000; Gage, 1979; Gibbs, 1978
Montacuta substriata	S	C	*Spatangus purpureus, Echinocardium flavescens* and other spatangoids	Attached to host	Gage, 1966; Fox *et al.*, 2007; Kallonas *et al.*, 1999
Montacutella echinophila	S	C	*Brissus latecarinatus*	Attached to host	Jespersen *et al.*, 2004
Montacutona ceriantha	S	C	*Cerianthus sp.*	Within host tubes	Ponder, 1971
Montacutona compacta	H	F		Attached to coral heads	Morton, 1980
Montacutona olivacea	S	C	*Cerianthus cf. filiformis*	Within host tubes	Morton, 1980
Mysella charcoti	S	F		Upper sediment layer	Domaneschi *et al.*, 2002; Passos *et al.*, 2005
Mysella cuneata	S	C	*Phascolion strombi*	Within host-occupied shells	Gage, 1979
Mysella gregaria	S	C	*Burowing actinian*	Attached to host	Rotvit *et al.*, 2007
Mysella narchii	S	F		Upper sediment layer	Passos and Domaneschi, 2006
Mysella pedroana	S	C	*Blepharipoda occidentalis; Isocheles pilosus*	Attached to host	Carpenter, 2005; Boyko and Mikkelsen, 2002
Mysella vitrea	S	C	*Trypaea australiensis;*	In host burrows	Kerr and Corfield, 1998;
Mysella vitrea	S	F	*Trypaea australiensis;*	In host burrows	Kerr and Corfield, 1998;
Neaeromya rugifera	S	C	*Upogebia pugettensis; Aphrodita sp.*	Attached to host	Boss, 1965b; Narchi, 1969; Ó Foighil, 1985
Nipponomysella subtruncata	S	C	*Siphonosoma cumanense*	Attached to host	Lützen *et al.*, 2001
Parabornia palliopapillata	S	C	*Lysiosquilla scabricauda*	Attached to host	Simone, 2001
Parabornia squillina	S	C	*Lysiosquilla scabricauda*	Attached to host	Boss, 1965
Peregrinamor ohshimai	S	C	*Upogebia major*	Attached to host	Itani *et al.*, 2002; Kato and Itani, 1995
Phlyctaenachlamys lysiosquillina	S	C	*Lysiosquillina maculata*	In host burrows	Popham, 1939
Pristes oblongus	H	C	Chitons		Kelsey, 1902
Pseudogaleomma japonica	H	F		Under rocks	Lützen and Nielsen, 2005; Ueng and Wang, 1999
Pseudopythina macrophthalmensis	S	C	*Macrophthalmus convexus*	Attached to host	Jespersen et al., 2001; Kosuge and Itani, 1994; Morton and Scott, 1989
Pseudopythina muris	S	C	*Aphtodita japonica*	Within host respiratory cavity	Rosewaer, 1984
Pseudopythina nodosa	S	C	*Sipunculus nudus*	Attached to host	Morton and Scott, 1989
Pseudopythina ochetostomae	S	C	*Ochetostoma erythrogrammon*	In host burrows	Jespersen *et al.*, 2002; Morton and Scott, 1989
Pseudopythina subsinuata	S	C	*Squilla nepa; Squilla raphidea; Oratosquilla oratorio*	Attached to host	Appukuttan 1972; Jespersen *et al.*, 2009; Morton 1972; Morton and Scott, 1989
Pseudopythina tsurumaru	S	C	*Protankyra bidentata*	Attached to host	Lützen *et al.*, 2004; Morton and Scott, 1989
Rochfortia (Mysella) tumida	S	C	*Mesochaetopterus taylori*	Within host's exhalent oxic halo	Sendall *et al.*, 1995
Scacchia oblonga	H	F		Within algal holdfasts	Kallonas *et al.*, 1999
Scintilla agilis	H	F		Under stones, within coral galleries	Lützen and Nielsen, 2005

Scintilla cuvieri	H	F		Under stones, within coral galleries	Lützen and Nielsen, 2005; Morton and Scott, 1989
Scintilla dubia	H	F		Under coral and slate blocks	Lützen and Nielsen, 2005
Scintilla imperatoris	H	F		Under dead coral	Lützen and Nielsen, 2005
Scintilla larcombae	H	F		In coral rubble crevices	Oliver and Holmes, 2004
Scintilla longitentaculata	H	F		Under stones	Lützen and Nielsen, 2005
Scintilla lynchae	H	F		Under coral and volcanic rock blocks	Oliver and Holmes, 2004
Scintilla macrodactylus	H	F		Under coral blocks	Lützen and Nielsen, 2005
Scintilla minor	H	F		Under coral blocks	Lützen and Nielsen, 2005
Scintilla mortoni	H	F		Under coral blocks	Lützen and Nielsen, 2005
Scintilla nitidella	H	F		Under coral, shale or rock blocks	Lützen and Nielsen, 2005
Scintilla nitidella	H	F		Under coral, shale or rock blocks	Lützen and Nielsen, 2005
Scintilla ovalis	H	F		Under rocks	Lützen and Nielsen, 2005
Scintilla ovulina	H	F		In coral galleries	Lützen and Nielsen, 2005
Scintilla papillosa	H	F		Under coral blocks	Lützen and Nielsen, 2005
Scintilla philippinensis	H	F		In crevices, under shale blocks	Lützen and Nielsen, 2005
Scintilla pisum	H	F		In coral rubble crevices	Oliver and Holmes, 2004
Scintilla sannio	H	F		Under rocks	Lützen and Nielsen, 2005
Scintilla (*Lactemiles*) *strangei*	H	F		Under rocks	Personal observation, Li, 2011
Scintilla unicornia	H	F		Under coral blocks	Lützen and Nielsen, 2005
Scintilla verrucosa	H	F		Under rocks	Lützen and Nielsen, 2005
Scintilla violescens	H	F		Attached to gorgonians	Arakawa, 1961; Kuroda and Taki, 1961
Scintilla vitrea	H	F		Under coral and volcanic rock blocks	Oliver and Holmes, 2004
Scintillona bellerophon	S	C	*Leptosynapta clarki*	Attached to host	Ó Foighil and Gibson, 1984
Scintillona brissae	S	C	*Brissus latecarinatus*	Attached to host	Jespersen *et al.*, 2004; Morton and Scott, 1989
Scintillona zelandica	S	C	*Trochodota dendyi*	Attached to host	Morton, 1957
Tellimya ferruginosa	S	C	*Echinocardium cordatum* and other spatangoids	In host burrows	Fox *et al.*, 2007; Gage, 1966; Kallonas *et al.*, 1999; Morton, 1962
Tellimya tenella	S	C	*Brissopsis lyrifera*	Attached to host	Fox *et al.*, 2007; Kallonas *et al.*, 1999
Varotoga cryptozoica (Scintilla anomala)	H	F		Undersides of stones, coral galleries	Lützen and Nielsen, 2005
Waldo parasiticus	S	C	*Tripylus sp; Abatus cavernosus; Abatus agassizii; Abatus cordatus; Abatus bidens; Triphylaster philippii; Triphylus excavatus*	Attached to host	Zelaya and Ituarte, 2002
Waldo trapezialis	S	C	Irregular echinoids	Attached to host	Zelaya and Ituarte, 2002

References

1. Altnöder A, Haszprunar G (2008) Larval morphology of the brooding clam *Lasaea adansonii* (Gmelin, 1791) (Bivalvia, Heterodonta, Galeommatoidea). Journal of Morphology 269: 762–774.

2. Appukuttan K (1972) *Pseudopythina subsinuata* (Lischke), a commensal bivalve of *Squilla nepa* (Latricelle) and *Squilla raphidea* Fabricius. Journal of the Marine Biological Association of India 14: 412–415.

3. Arakawa K (1960) Ecological observations on an aberrant lamellibranch, *Ephippodonta murakamii*. Venus 21: 50–61.

4. Arakawa K (1961) A note on the animal of *Scintilla violescens* collected in Genkai Sea. Venus 21: 143–146.

5. Boss K (1965) Symbiotic erycinacean bivalves. Malacologia 3: 183-195.

6. Boss K (1965) A new mollusk (Bivalvia, Erycinidae) commensal on the stomatopod crustacean *Lysiosquilla*. American Museum Novitates 2215: 1-11.

7. Bourne G (1906) On *Jousseaumia*. A new genus of eulamellibranchs commensal with the corals *Heterocyathus* and *Heteropsammia*. Ceylon Pearl Oyster Fisheries Supplemntary Reports No. XXXVII: 212-266.

8. Boyko CB, Mikkelsen PM (2002) Anatomy and biology of *Mysella pedroana* (Mollusca: Bivalvia: Galeommatoidea), and its commensal relationship with *Blepharipoda occidentalis* (Crustacea: Anomura: Albuneidae). Zoologischer Anzeiger - A Journal of Comparative Zoology 241: 149–160.

9. Bristow GA, Berland B, Schander C, Vo DT (2010) A new endosymbiotic bivalve (Heterodonta: Galeommatoidea), from Pacific holothurians. The Journal of Parasitology 96: 532–534.

10. Carpenter S (2005) *Mysella pedroana*, a commensal bivalve (Lasaeidae) on two decapod crustacean hosts. Nautilus 119: 105–108.

11. Chanley P, Chanley M (1970) Larval development of the commensal clam, *Montacuta percompressa* Dall. Proceedings of the Malacological Society of London 39: 59–67.

12. Chanley P, Chanley M (1980) Reproductive biology of *Arthritica crassiformis* and *A. bifurca*, two commensal bivalve molluscs (Leptonacea). New Zealand Journal of Marine and Freshwater Research 14: 31–43.

13. Clench W, Aguayo C (1931) *Entovalva* (*Devonia*) *perrieri* (Malard) in the Western Atlantic. Occasional Papers of the Boston Society of Natural History 8: 5-8.

14. Cotton BC (1938) *Ephippodonta* – South Australia's most perculiar bivalve shell. Victorian Naturalist 55: 58–61.

15. Crisp DJ, Standen A (1988) *Lasaea rubra* (Montagu) (Bivalvia: Erycinacea), an apomictic crevice-living bivalve with clones separate by tidal level preference. Journal of Experimental Biology and Ecology 117: 27-45.

16. Domaneschi O, da Silva J, Neto L, Passos F (2002) New perspectives on the dispersal mechanisms of the Antarctic brooding bivalve *Mysella charcoti* (Lamy, 1906). Polar Biology 25: 538–541.

17. Ó Foighil D, Gibson A (1984) The morphology, reproduction and ecology of the commensal bivalve *Scintillona bellerophon* spec. nov (Galeommatacea). Veliger 27: 72–80.

18. Ó Foighil D, Thiriot-Quievreux C (1999) Sympatric Australian *Lasaea* species (Mollusca : Bivalvia) differ in their ploidy levels, reproductive modes and developmental modes. Zoological Journal of the Linnean Society 127: 477–494.

19. Fox TH, Jespersen Å, Lützen J (2007) Sperm transfer and reproductive biology in species of hermaphroditic bivalves (Galeommatoidea: Montacutidae). Journal of Morphology 268: 936–952.

20. Gage J (1966) Experiments with the behaviour of the bivalves *Montacuta substriata* and *M. ferruginosa*, 'commensals' with spatangoids. Journal of the Marine Biological Association of the United Kingdom 46: 71–88.

21. Gage J (1979) Mode of life and behaviour of *Montacuta phascolionis*, a bivalve commensal with the sipunculan *Phascolion strombi*. Journal of the Marine Biological Association of the United Kingdom 59: 635–657.

22. Gibbs PE (1978) *Menestho diaphana* (Gastropoda) and *Montacuta Phascolionis* (Lamellibranchia) in association with the sipunculan *Phascolion strombi* in Brsitish Waters. Journal of the Marine Biological Association of the United Kingdom 58: 683-685.

23. Gofas S (1991) The family Galeommatidae (Bivalvia: Leptonacea) in the Eastern Atlantic. Veliger 34: 344–353.

24. Gofas S, Salas C (2008) A review of European '*Mysella*' species (Bivalvia, Montacutidae), with description of *Kurtiella* new genus. Journal of Molluscan Studies 74: 119–135.

25. Goto R, Hamamura Y, Kato M (2007) Obligate commensalism of *Curvemysella paula* (Bivalvia: Galeommatidae) with hermit crabs. Marine Biology 151: 1615–1622.

26. Itani G, Kato M, Shirayama Y (2002) Behaviour of the shrimp ectosymbionts, *Peregrinamor ohshimai* (Mollusca : Bivalvia) and *Phyllodurus sp.* (Crustacea : Isopoda) through host ecdyses. Journal of the Marine Biological Association of the United Kingdom 82: 69-78.

27. Iwasaki K (1996) Seasonal changes in size structure and reproduction of the minute Galeommatacean Bivalve *Lasaea undulata* (Gould) within intertidal mussel beds. The Veliger 39: 244-249.

28. Jespersen Å, Lützen J, Oliver PG (2007) Morphology, biology and systematic position of *Epilepton clarkiae* (Clark, 1852)(Galeommatoidea: Montacutidae) a bivalve commensal with sipunculans. Journal of Conchology 39: 391-401.

29. Jespersen Å, Lützen J (2000) Sex, seminal receptacles, and sperm ultrastructure in the commensal bivalve *Montacuta phascolionis* (Veneroida; Galeommatacea). Acta Zoologica 81: 69–75.

30. Jespersen Å, Lützen J (2001) Ultrastructure of the seminal receptacle and the dimorphic sperm in the commensal bivalve *Mysella bidentata* (Veneroida; Galeommatoidea; Montacutidae). Acta Zoologica 82: 107–115.

31. Jespersen Å, Kosuge T, Lützen J (2001) Sperm dimorphism and spermatozeugmata in the commensal bivalve *Pseudopythina macrophthalmensis* (Galeommatoidea, Kelliidae). Zoomorphology 120: 177–189.

32. Jespersen Å, Lützen J, Morton B (2002) Ultrastructure of dimorphic sperm and seminal receptacle in the hermaphrodites *Barrimysia siphonosomae* and *Pseudopythina ochetostomae* (Bivalvia, Galeommatoidea). Zoomorphology 121: 159–172.

33. Jespersen Å, Lützen J, Nielsen C (2004) On three species and two new genera (*Montacutella* and *Brachiomya*) of galeommatoid bivalves from the irregular sea urchin *Brissus latecarinatus* with emphasis on their reproduction . Zoologischer Anzeiger 243: 3–19.

34. Jespersen Å, Lützen J (2007) Sperm ultrastructure in *Kellia suborbicularis* (Bivalvia : Galeommatoidea : Kellidae). Acta Zoologica 88: 59–63.

35. Jespersen Å, Lützen J, Itani G (2009) Sperm structure and sperm transfer in *Pseudopythina subsinuata* (Bivalvia; Galeommatoidea). Zoologischer Anzeiger 248: 57–67.

36. Judd W (1971) The structure and habits of *Divariscintilla maoria* Powell (Bivalvia: Galeommatidae). Journal of Molluscan Studies 39: 343-354.

37. Kallonas M, Zenetos A, Gofas S (1999) Notes on the ecology and distribution of microbivalvia in Greek waters. La Conchiglia 291: 11-20.

38. Kato M, Itani G (1995) Commensalism of a bivalve, *Peregrinamor ohshimai*, with a thalassinidean burrowing shrimp, *Upogebia major*. Journal of the Marine Biological Association of the United Kingdom 75: 941–947.

39. Kato M (1998) Morphological and ecological adaptations in montacutid bivalves endo- and ectosymbiotic with holothurians. Canadian Journal of Zoology 76: 1403–1410.

40. Kawahara T (1942) On *Devonia oshimai* sp. nov., a commensal bivalve attached to the synaptid *Leptosynapta ooplax*. Venus 11: 153–164.

41. Keep J, Hannibal HB (1911) West Coast Shells. A description of the principal marine mollusks living on the west coast of the United States, and of the land shells of the adjacent region. San Francisco: The Whitaker & Ray-Wiggin Company.

42. Kerr G, Corfield J (1998) Association between the ghost shrimp *Trypaea australiensis* Dana 1852 (Crustacea : Decapoda) and a small deposit-feeding bivalve *Mysella vitrea* Laserson 1956 (Mollusca: Leptonidae). Marine and Freshwater Research 49: 801–806.

43. Kosuge T, Itani G (1994) A record of the crab associated bivalve, *Pseudopythina macrophthalmensis* from Iriomote Island, Okinawa, Japan. Venus 53: 241-244.

44. Kubo H (1996) *Ephippodonta gigas* n. sp. (Bivalvia: Galeommatoidea) from Okinawa Island, Southwestern Japan. Venus 55: 1-5.

45. Kuroda T, Taki I (1961) On a new species of *Scintilla* (Galeommatidae) from Japan. Venus 21: 141-142.

46. Lamy ME (1908) Description d'une coquille nouvelle de la Côte Atlantique Française. Journal de Conchyliologie 56: 35-37.

47. Laseron CF (1956) A revision of the New South Wales Leptonidae. Records of the Australian Museum 24: 7–22.

48. Lebour M (1938) The life history of *Kellia suborbicularis*. Journal of the Marine Biological Association of the United Kingdom 22: 447-451.

49. Lützen J, Takahashi T, Yamaguchi T (2001) Morphology and reproduction of *Nipponomysella subtruncata* (Yokoyama), a galeommatoidean bivalve commensal with the sipunculan *Siphonosoma cumanense* (Keferstein) in Japan. Journal of Zoology 254: 429–440.

50. Lützen J, Takahashi T (2003) *Arthritica japonica*, sp. nov.(Bivalvia: Galeommatoidea: Leptonidae), a commensal with the pinnotherid crab *Xenophthalmus pinnotheroides* White, 1846. Yuriyagai 9: 11–19.

51. Lützen J, Jespersen Å, Takahashi T, Kai T (2004) Morphology, structure of dimorphic sperm, and reproduction in the hermaphroditic commensal bivalve *Pseudopythina tsurumaru* (Galeommatoidea : Kellidae). Journal of Morphology 262: 407–420.

52. Lützen J, Kato M, Kosuge T, Ó Foighil D (2005) Reproduction involving spermatophores in four bivalve genera of the superfamily Galeommatoidea commensal with holothurians. Molluscan Research 25: 99-112.

53. Lützen J, Nielsen C (2005) Galeommatid bivalves from Phuket, Thailand. Zoological Journal of the Linnean Society 144: 261–308.

54. Lützen J, Hong J, Yamashita H (2009) *Koreamya arcuata* (A. Adams, 1856) gen. nov.(Galeommatoidea: Montacutidae), a commensal bivalve associated with the inarticulate brachiopod *Lingula anatina*. Journal of Conchology 39: 669.

55. Manning RB, Morton B (1987) Pinnotherids (Crustacea: Decapoda) and Leptonaceans (Mollusca: Bivalvia) associated with sipunculan worms in Hong Kong. Proceedings of the Biological Society of Washington 100: 543-551.

56. Middelfart P, Craig M (2004) Description of *Austrodevonia sharnae* n. gen. n. sp. (Galeommatidae: Bivalvia), an ectocommensal of *Taeniogyrus australianus* (Stimpson, 1855)(Synaptidae: Holothuroidea). Molluscan Research 24: 211–219.

57. Middelfart P (2005) Review of *Ephippodonta sensu lato* (Galeommatidae: Bivalvia), with descriptions of new related genera and species from Australia. Molluscan Research 25: 129–144.

58. Mikkelsen PM, Bieler R (1989) Biology and comparative anatomy of *Divariscintilla yoyo* and *D. troglodytes*, two new species of Galeommatidae (Bivalvia) from stomatopod burrows in eastern Florida. Malacologia 31: 175–195.

59. Mikkelsen PM, Bieler R (1992) Biology and comparative anatomy of three new species of commensal Galeommatidae, with a possible case of mating-behavior in bivalves. Malacologia 34: 1–24.

60. Morton JE (1957) The habits of *Scintillona zelandica* (Odhner) 1924 (Lamellibrancma: Galeommatidae). Proceedings of the Malacological Society 32: 185-188.

61. Morton B (1962) Habit and orientation in the small commensal bivalve mollusc, *Montacuta ferruginosa*. Animal Behaviour 10: 126-133.

62. Morton B (1972) Some aspects of functional morphology and biology of *Pseudopythina subsinuata* (Bivalvia: Leptonacea) commensal on stomatopod crustaceans. Journal of Zoology 166: 79-96.

63. Morton B (1973) Some factors affecting location of *Arthritica crassiformis* (Bivalvia: Leptonacea) commensal upon *Anchomasa similis* (Bivalvia: Pholadidae). Journal of Zoology 170: 463–473.

64. Morton B (1973) Biology and functional morphology of *Galeomma (Paralepida) takii* (Bivalvia: Leptonacea). Journal of Zoology 169: 133–150.

65. Morton B (1980) Some aspects of the biology and functional morphology (including the presence of a ligamental lithodesma) of *Montacutona compacta* and *Montacutona olivacea* (Bivalvia, Leptonacea) associated with coelenterates in Hong Kong. Journal of Zoology 192: 431–455.

66. Morton B (1981) The biology and functional morphology of *Chlamydoconcha orcutti* with a discussion on the taxonomic status of the Chlamydoconchacea (Mollusca: Bivalvia). Journal of Zoology 195: 81–121.

67. Morton B, Valentich-Scott P (1989) The Hong Kong Galeommatacea (Mollusca: Bivalvia) and their hosts, with descriptions of new species. Asian Marine Biology 6: 129–160.

68. Narchi W (1969) On *Pseudopythina rugifera* (Carpenter, 1864) (Bivalvia). Veliger 12: 43-52.

69. Nicell L, Atkinson R, Hughes D, Ansell A, Smith C (1995) Burrow morphology of the echiuran worm *Maxmuelleia Lankesteri* (Echiura, Bonelliidae), and a brief review of burrow structure and related ecology of the Echiura. Journal of Natural History 29: 871–885.

70. Norman A (1891) *Lepton squamosum* (Montagu), a commensal. The Annals and Magazine of Natural history 7: 276-278.

71. Ockelmann KW, Muus K (1978) The biology, ecology, and behavior of the bivalve *Mysella bidentata* (Montagu). Ophelia 17: 1–93.

72. Ó Foighil D (1985) Form, function, and origin of temporary dwarf males in *Pseudopythina rugifera* (Carpenter, 1864)(Bivalvia: Galeommatacea). Veliger 27: 72-80.

73. Ohshima H (1931) On *Entovalva semperi* Ohshima, an aberrant commensal bivalve. Venus 2: 161–177.

74. Oliver PG (1993) A new commensal bivalve associated with a burrowing sea anemone *Halcampicola tenacis* gen. *et* sp. nov., (Galeommatoidea: Montacutidae) on *Halcampoides sp.* (Anthozoa: Antipatharia: Halcampoididae). In: Wells FE, Walker DI, Kirkman H, Lethbridge R, editors, Proceedings of the Fifth International Marine Biological Workshop: The Marine Flora and Fauna of Rottnest Island, Western Australia. Perth: Western Australian Museum, p. 634.

75. Oliver PG, Holmes AM, Killeen IJ, Turner JA (2010). Marine Bivalve Shells of the British Isles (Mollusca: Bivalvia).

76. Passos F, Domaneschi O, Sartori AF (2005) Biology and functional morphology of the pallial organs of the Antarctic bivalve *Mysella charcoti* (Lamy, 1906) (Galeommatoidea: Lasaeidae). Polar Biology 28: 372–380.

77. Passos FD, Domaneschi O (2006) A new species of *Mysella Angas*, 1877 (Bivalvia: Galeommatoidea) from Admiralty Bay, King George Island, South Shetlands, Antarctica, with data on its biology and functional anatomy. Polar Biology 29: 389–398.

78. Ponder WF (1965) The biology of the genus *Arthritica*. Transactions of the Royal Society, New Zealand, Zoology, 6: 75–86.

79. Ponder WF (1971) Some New Zealand and Subantarctic bivalves of the Cyamiacea and Leptonacea with descriptions of new taxa. Records of the Dominion Museum 7: 119-141.

80. Ponder WF (1971) '*Montacutona ceriantha*' n. sp., A commensal leptonid bivalve living with 'cerianthus'. Journal de Conchyliologie 109: 16-25.

81. Popham ML (1939) On *Phlyctaenachlamys lysiosquillina* gen. *et* sp. nov: a lamellibranch commensal in the burrows of *Lysiosquilla maculara*. British Museum, Natural History, Great Barrier Reef Expedition, 1928-29 Scientific Reports 6: 61–84.

82. Rosewater J (1984) A new species of leptonacean bivalve from off Northwestern Peru (Heterodonta, Veneroida, Lasaeidae). Veliger 27: 81–89.

83. Rotvit L, Lützen J, Jespersen Å, Fox T (2007) *Mysella gregaria* new species, a bivalve (Galeommatoidea : Montacutidae) commensal with an intertidal burrowing sea anemone from North Carolina, USA. Nautilus 121: 191–200.

84. Sato S, Owada M, Haga T, Hong JS, Lützen J, et al. (2011) Genus-specific commensalism of the galeommatoid bivalve *Koreamya arcuata* (A. Adams, 1856) associated with lingulid brachiopods. Molluscan Research 31: 95–105.

85. Simone (2001) Revision of the genus *Parabornia* (Bivalvia: Galeommatoidea: Galeommatidae) from the Western Atlantic with description of a new species from Brazil. Journal of Conchology 37: 159–169.

86. Spärck R (1931) *Cycladoconcha amboiensis* n. gen. n. sp. A commensalistic lamellibranch. Papers from Dr. Th. Mortensens's Pacific Expedition 1914-16. Videnskabelige Meddelelser Dansk Naturhis torisk Forening 91: 227–239.

87. Ueng YT, Wang JP (1999) *Pseudogaleooma japonica* (Galeommatidae), a familial and generic record new to Veneroida fauna of Taiwan. Journal of Taiwan Museum 52: 7-11.

88. Voeltzkow A (1890) *Entovalva mirabilis*, eine schmarotzende Muschel aus dem Darm einer Holothurie. Zoologische Jahrbücher, Abteilung für Systematik, Ökologi und Geographie der Tiere 5: 619–628.

89. Yamamoto T, Habe T (1974) *Scintillona stigmatica* new to Japan. Venus 33: 115-116.

90. Zelaya DG, Ituarte C (2002) The identity of *Waldo parasiticus* (Dall, 1876) and description of *Waldo trapezialis* new species (Bivalvia: Galeommatoidea). Nautilus 116: 109–117.
